# A Flexible Autonomous Robotic Observatory Infrastructure for Bentho-Pelagic Monitoring †

**DOI:** 10.3390/s20061614

**Published:** 2020-03-13

**Authors:** Jacopo Aguzzi, Jan Albiez, Sascha Flögel, Olav Rune Godø, Endre Grimsbø, Simone Marini, Olaf Pfannkuche, Erik Rodriguez, Laurenz Thomsen, Terje Torkelsen, Javier Valencia, Vanesa López-Vázquez, Henning Wehde, Guosong Zhang

**Affiliations:** 1 Instituto de Ciencias del Mar (ICM-CSIC), E-08003 Barcelona, Spain; jaguzzi@icm.csic.es; 2 Stazione Zoologica Anton Dohrn, 80122 Naples, Italy; 3 Kraken Robotik, 28197 Bremen, Germany; jalbiez@krakenrobotik.de; 4 GEOMAR, Helmholtz Centre for Ocean Research Kiel, 24148 Kiel, Germany; sfloegel@geomar.de; 5 Metas AS, N-5106 Bergen, Norway; terjet@metas.no; 6 Norwegian Research Centre NORCE, N-5838 Bergen, Norway; 7 Institute of Marine Research, N-5817 Bergen, Norway; endreg@hi.no (E.G.); henningw@hi.no (H.W.); guosong.zhang@hi.no (G.Z.); 8 The Arctic University of Norway, N-9037 Tromsø, Norway; 9 CNR-ISMAR, 19032 La Spezia, Italy; simone.marini@sp.ismar.cnr.it; 10 iSeaMC, 28759 Bremen, Germany; o.pfannkuche@iseamc.com (O.P.); l.thomsen@iseamc.com (L.T.); 11 DS LABS, 01015 Vitoria-Gasteiz, Spain; erodriguez@deustosistemas.net (E.R.); vlopez@deustosistemas.net (V.L.-V.); 12 Jacobs University, 28759 Bremen, Germany; 13 Deusto Sistemas, E-01015 Vitoria-Gasteiz, Spain; javi.valencia.m@gmail.com

**Keywords:** benthic and pelagic monitoring, image processing, acoustics, crawler, docking station, cabled observatories, fuel cells, ecosystem component classification

## Abstract

This paper presents the technological developments and the policy contexts for the project “Autonomous Robotic Sea-Floor Infrastructure for Bentho-Pelagic Monitoring” (ARIM). The development is based on the national experience with robotic component technologies that are combined and merged into a new product for autonomous and integrated ecological deep-sea monitoring. Traditional monitoring is often vessel-based and thus resource demanding. It is economically unviable to fulfill the current policy for ecosystem monitoring with traditional approaches. Thus, this project developed platforms for bentho-pelagic monitoring using an arrangement of crawler and stationary platforms at the Lofoten-Vesterålen (LoVe) observatory network (Norway). Visual and acoustic imaging along with standard oceanographic sensors have been combined to support advanced and continuous spatial-temporal monitoring near cold water coral mounds. Just as important is the automatic processing techniques under development that have been implemented to allow species (or categories of species) quantification (i.e., tracking and classification). At the same time, real-time outboard processed three-dimensional (3D) laser scanning has been implemented to increase mission autonomy capability, delivering quantifiable information on habitat features (i.e., for seascape approaches). The first version of platform autonomy has already been tested under controlled conditions with a tethered crawler exploring the vicinity of a cabled stationary instrumented garage. Our vision is that elimination of the tether in combination with inductive battery recharge trough fuel cell technology will facilitate self-sustained long-term autonomous operations over large areas, serving not only the needs of science, but also sub-sea industries like subsea oil and gas, and mining.

## 1. Introduction

New technology has always been the driver for breakthroughs in marine ecology. When ecosystems exploration and monitoring are enforced in the deep-sea realms, spectacular development in robotics emerges [1]. Now, mechatronics and the Internet of Things paradigm are setting the basis for the development of improved platform mobility and autonomy in navigation and sampling [2,3]. In this scenario, cabled observatories and their infrastructures are integrated with spatial mobile networks where the fixed nodes coordinate the functioning of movable platforms (e.g., docked Remotely Operated Vehicles (ROVs) and Autonomous Underwater Vehicles (AUVs)) in-between [4]. Such development will promote the spatially extensive and temporally intensive monitoring of ecosystem biodiversity and functioning in relation to, for example, the rate and quantity of energy exchanged within the food web and resulting biomass production with special reference to management services for exploited fish stocks. We also note that the turnover of marine life in deep-sea benthic communities is slow with sometimes extremely long recovery time [5]. Any sampling tool and associated sampling design must therefore be planned to minimize the damaging risk on the fragile parts of the ecosystem. The overall goal is the reduction of vessel-assisted operations, which have very high operating costs, by enhancing platforms operability and autonomy in situ. In this framework, optoacoustic imaging technologies play a central role in determining richness and the relative abundance of species within a wide range of ecological sizes (e.g., [6]), delivering at the same time life history trait data relevant to management and conservation (e.g., phenology such as activity rhythms, growth and reproduction cycles, ecological interactions, bioturbation) [7].

In this paper, we present the technological solutions implemented to enable remote, autonomous, and long-lasting monitoring of the deep-sea ecosystem. Demonstration and development are done in a Norwegian coastal deep-sea canyon with cold water coral (CWC) at approximately 250 m depth within the framework of the project, Autonomous Robotic Sea-Floor Infrastructure for Bentho-Pelagic Monitoring (ARIM). This project aims to deliver new advanced robotic products for deep-sea bentho-pelagic monitoring on representative ecological scales (i.e., from a few meters around cabled nodes to hundreds of meters around). A new prototype of a mobile crawler and cabled observatory with an integrated docking station, from which it can be operated, has been designed. At the same time, the autonomy of the system with novel fuel cell technology is under development. Our vision is that fuel cell technology will expand the use of crawlers in self-sustained long-term autonomous operations, for science as well as for industry purposes, when the umbilical is eventually removed.

The first step in development is centered on advancing crawler autonomy under controlled conditions (i.e., with a tether to explore the neighborhood of the stationary instrumented garage). A combination of visual and acoustic sensing along with standard oceanographic sensors has been conceived to support remote, high-frequency, and continuous multiparametric biological and environmental data collection and on-board data processing. A core activity is therefore to develop artificial intelligence (AI) algorithms that facilitate processing of the images required for automated adaptive driving as well as for the identification of biological and habitat characteristics (i.e., counted individuals and species local abundances, coral growth). This development aim at supporting science and monitoring services without backing from expensive ship time. The objectives of this paper are to describe the technology and ecological basis for the development of ARIM as well as demonstrate its applicability for research and industry through generated results; the novelty of our work is linking the various technologies together in an interdisciplinary fashion.

## 2. Materials and Methods

Cabled marine observatories are developed worldwide and will eventually become an important component of global marine monitoring networks [1,8,9,10]. In Section 2.1, we present the Norwegian deployment area and already existing cabled observatory infrastructures, within which ARIM is being developed. Then, we describe the technology components and the associated tasks (Section 2.2 and Section 2.3). This includes the design of the ARIM concept, and how all components are tailored for the project purpose and turned into a coherent integrated product for research and monitoring.

### 2.1. The Hosting Infrastructure and the Development Area

**Figure 1:**
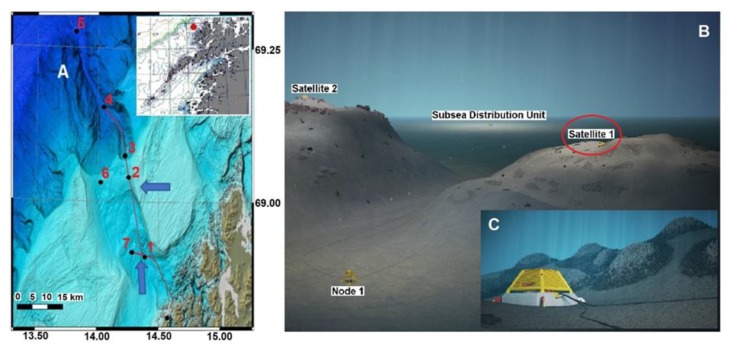
Overview of the study area where the Lofoten-Vesterålen (LoVe) observatory is located. (**A**) The bathymetric map of the canyon area with the extended cabled transect with the nodes (black dots) numbered 1–7 and connected by a telecommunication cable (continuous line). Node 1 is located in the deeper part of the trough (marked with southernmost blue arrow). This is a central location for *Lophelia* reef mounds. The new Autonomous Robotic Sea-Floor Infrastructure for Bentho-Pelagic Monitoring (ARIM) test platform will be located at node 2 (northernmost blue arrow). (**B**) A three-dimensional (3D) detailed representation of the area around node 1, where the satellite X-frame with video camera is encircled (in red). Here we collected the footage used to establish the artificial intelligence (AI) procedures for later transfer into the crawler for on-board image autonomous processing. (**C**) Enlarged view of the areas surrounding the node where *Lophelia* reefs are schematized.

The Norwegian continental shelf hosts some of the richest CWC reefs in the world, which act as shelters and nurseries for a number of species of economic interest [11]. To better understand their biological and physical settings, cabled observatory nodes were installed in the Hola trough (Figure 1), a deep-sea area with CWC mounds surrounded by complex oceanographic conditions, off the Norwegian coast [8,12]. This was stage one of the development of the Lofoten-Vesterålen (LoVe) ocean observatory [13], which has been in operation since 2013 [8].

The full LoVe infrastructure consists of a high voltage (3 kV) backbone power cable with fiber-optic communication and several 240V, 50Hz connection points including subsea distribution units (SDUs); see Section 3.2. The full system consists of several nodes connected to the infrastructure, with instrumentation and sensor technology for measuring environmental parameters and processes related to oceanography, geology, and biology. Measuring equipment and nodes are supplied by several manufacturers, including Metas. The X-frame, part of the Metas X-net [14] (Figure 1, see also Section 3.2), has for several years been the basic central unit collecting data either from sensors installed on the X-frame or from sensors mounted at the satellite platforms (Figure 1). The X-frame is directly connected to the SDUs for power and communication.

LoVe is constructed with a robust and flexible infrastructure that can accommodate additional observation/sampling platforms and sensors that meet different ecosystem monitoring requirements. The observatory network is a good example of joint science-industry-based ecosystem monitoring efforts, of relevance for many industries (i.e., petroleum, fisheries), although the objectives might be potentially conflicting. The observatory is based on technical requirements that meet the North Sea standard defined for the petroleum industry. This represents higher initial investments but lower operational costs in the long run. The experience so far has been positive with relatively stable operation of multiple sensor platforms including echosounders, oceanographic sensors, and high-definition (HD) cameras.

Node 1 is located at a depth of ∼260 m, 20 km off the coast north of the Lofoten Islands (Norway) in the Hola trough (see Figure 1). This glacially-created trough substantially increases the continental shelf depth in a north-west to south-east direction. The location of node 1 is enclosed by two 100-m deep banks: Vesterålsgrunnen in the northeast and Eggagrunnen in the southwest. The trough has a diverse topography with sand wave fields of up to 7 m high, 10–35 m high ridges, and approximately 20 m high CWC mounds [15]. The CWC mounds are predominantly found in the south-eastern part of the trough at a depth of ∼260 m, just south of the Vesterålsgrunnen bank, and are mostly constituted by CWC *Lophelia pertusa*. A satellite with a camera (see Figure 1) provided data for the AI training during the first year of ARIM (see Section 2.2).

The LoVe observatory is now under expansion and 7 nodes will cover the continental shelf and the shelf break, with depths ranging from about 100 m down to 2500 m (see Figure 1). Nodes 1, 3, 4, and 7 are connected to the backbone cable; node 5 and 6 are autonomous with the option for acoustic communication to backbone nodes. All nodes are now implemented, except node 2 and 6, but some have incomplete instrumentation and functionality. Data from this expansion will enhance understanding of the interaction between offshore Atlantic water and inshore coastal water. Further, transect data support in understanding of the impact of water circulation on the ecosystem in general and on harvested fish stocks, in particular, that spawn in this area [8]. The new ARIM platform with the crawler will be tested connected to node 2, west of the original observatory (see Figure 1).

At an early stage, the involved scientists and engineers realized that their original goals were unobtainable by only one of the partners. This became the basis for the wide international partnership, as apparent from the list of authors and institutions specialized in robotic developments and ecology. Our motivation for this approach is based on three main principles:

1. Collaboration between science and industry creates substantial research and development (R&D) gains;
2. No research or industry organization alone holds the competence and knowledge needed to develop usable robotic tools for sustainable management of the marine environment and its resources;
3. Multidisciplinary and multi-nationality cooperation fosters new ideas and solutions that are applicable to an extended market of industrial maritime users. In the future, ecological monitoring will be requested from these industries to demonstrate the sustainability of their activity.

### 2.2. AI Developments for Image Processing

While autonomy in space [16] and terrestrial robots have experienced significant increases in research and applied technologies, robots in the underwater domain are still mostly tele-operated, although this scenario is closest to space research [17]. Tethered crawler technology operated remotely through an Internet-based connection, has already been used in association with cabled observatory infrastructure [18,19]. The crawler is belt driven, which gives access to a great variety of habitats. When the crawler and its sensors are available via Ethernet communication, the piloting of the remotely operated vehicle is supported by online camera information. This combination facilitates an important fundament for the development of autonomy. Our new autonomous crawler solution now under testing, is equipped with camera and standard oceanographic sensors and is designed based on 10 years of experience. While tethered crawlers have so far mainly been tele-operated, new monitoring needs are pushing toward fully self-sustained independent navigation and data collection. The fundament for this development is on-board data collection and processing of visual and bathymetry information in near real-time. This requires advanced navigation algorithms, which are now under development.

Our solution includes a stepwise approach. The first step was to create supervised autonomous navigation, that allows the crawler to do more complex data gathering missions on its own, while its activities are monitored though cable communication. The next step is to operate the crawler fully autonomously without a cable. At the same time, autonomy in biological data collection is pursued to efficiently monitor megafauna (i.e., animals larger than 2 cm in size). In full operation, the system not only collects data but also extracts biological information on-board and presents results for the user. A major part of our development includes processing images in order to track and classify motile species of fishes, cephalopods, echinoderms, and crustaceans [20]; and in the case of long-term monitoring, establish information on activity patterns at diel and seasonal scales, as well as growth patterns of CWCs [4,7,18,21].

Below we describe the development from interactive to fully autonomous operation via a data-cabled connection for training the navigation and animals counting software. The specific technology and operational tasks include:

- Create a docking interface for ROV free maintenance with an intelligent mobile seafloor monitoring system (crawler; [22] to provide independence from an infrastructure cable;
- Drastically increase the operational area and capability of the crawler by using an extended fiber-optic cable and thus provide the option to operate without umbilical;
- Construct a deep-sea fuel cell to provide energy for long-term self-sustained operations;
- Improve observation capacities using state-of-the-art active acoustics and 3D camera technologies;
- Implement routines for automatic organism classification and tracking and integrate this information with the crawler sensor data;
- Improve integration of hardware/software components to provide an efficient user interface;
- Demonstrate performance and develop training facilities for new users and markets.

### 2.3. The Targeted Ecosystem Functioning Components and Monitoring Approach

Ecosystem functioning is the mode and rate of circulation of organic and inorganic matter among species within a community, and such studies require sensors deployed along benthic and pelagic environmental gradients [4,23]. Behavior and its spatiotemporal modulation cover an important role in that transference. Thus, when populations migrate at tidal, inertial, or day-night temporal scales, then they are active carriers of organic carbon into 3D water column seabed scenarios [24,25]. Their intermittent presence into different continental margins and abyssal depth strata influences the rate of their energy exchanges beyond classic benthopelagic and upwelling phenomena in a still poorly understood fashion. When high-frequency and multiannual image acquisition are coupled with concomitant environmental monitoring, solid cause–effect relationships can be established measuring the behavior of individuals in response to environmental cycles (i.e., the activity rhythms) and stochastic perturbations [10,21]. When that monitoring is replicated over observational platforms into a network, the validity of local observations can be extrapolated at larger spatial and ecological scales [1]. In this context, automatic image and data processing would fundamentally improve monitoring through the analysis of biological/environmental interrelated data complexly [1]. The envisaged ARIM development aims to target megafauna with imaging and acoustic technologies to measure behavioral rhythms of community components, including composition changes at both smaller (i.e., daily) and larger (i.e., seasonal, interannual) temporal scales. We aim to identify the best monitoring strategies and data processing routines to extrapolate platform data at larger spatial scales of the network, choosing the best multivariate statistic strategies to link the observed variability in animal abundances to variation in environmental variables.

## 3. Results: Progress of System Assembly and Data Production

**Figure 2:**
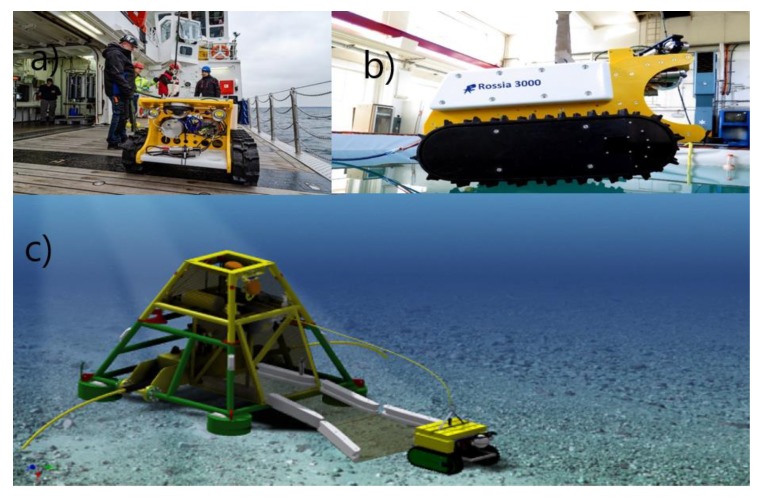
The ARIM benthic platform (X-Net^®^, Metas AS, Bergen, Norway) with the battery driven Rossia crawler. (**a**) and (**b**) show Rossia equipped with cameras and oceanographic sensors tested on-board R/V Alkor in November 2019 (**a**) and in laboratory (**b**). (**c**) shows the bottom platform (X-Frame and X-Node) with garage. (See Figure 3 for details about X-Net^®^ (Metas AS, Bergen, Norway)). During the field tests Rossia was equipped with the basic payload and remotely controlled at 10 m depth through a surface buoy.

This section describes the basic technology on which ARIM was developed and explains in detail the hardware (Section 3.1, Section 3.2 and Section 3.3) and software (Section 3.4 and Section 3.5) development done in the project and illustrates the outcome of the technology in terms of example results (Section 3.5). So far, we have experience with the various technologies separately. We now aim to merge cable-based observatory, mobile robotic seafloor technology images, and acoustic processing with modeling methods into one operational autonomous product (Figure 2). This required work along five avenues that in the end were combined into a coherent subsea monitoring tool.

### 3.1. The Crawler System

An early version of the crawler has been in operation in the Neptune observatory of Barkley canyon (off Victoria, B.C., Canada; from 2010 [26,27]. The robustness and stability demonstrate that this unit is ready to be used in routine monitoring systems. Based on the Neptune experience, an improved crawler version called “Rossia” (Figure 2) with a functionality tailored to the ARIM project, was constructed. Rossia has a plug-and-play interface facilitating easy implementation of new sensors according to the function and usage of the system. In the first version of ARIM the crawler was connected to the garage through an Ethernet cable without power. Navigation with the cable behind the vehicle without entangling it in habitat obstacles is a critical element in the functionality. We envisaged therefore two options: (i) installing a winch on the crawler, to pay out cable as the crawler moves. A cable heavier than water is left on the bottom until the crawler starts moving “home” along the exact same path. The winch will then start a tension-based retrieval of the cable. (ii) An alternative solution is a garage-based winch and a positive buoyant cable. Alternative (i) was considered safer but the winch consumes power and reduces operation duration autonomy. Alternative (ii) with a floating cable increases risk for cable abrasion and entanglement to the bottom habitat but extends operation in time and space. The key crawler sensor is the imaging system collecting information about the surveyed benthic habitat. Repeated surveys enable analysis of the development of the benthic marine life over time, where changes detected in the acquired data are associated to changes in the marine environment following monitoring protocols already developed in other studies [28,29,30,31,32]. The caterpillar tracks create a footprint on the seafloor of 0.35 m^2^ with a weight of ≈ 10 g/cm^2^, thus very little compaction and disturbance of sediments can be expected. Throughout typical operations during environmental monitoring the crawler stays on predetermined tracks during the transect analyses. The operator uses the cameras and the manipulator to record and probe sediments in the vicinity. This procedure is used to avoid unnecessary damage of the fragile environment [26].

### 3.2. The Bottom-Based System

**Figure 3:**
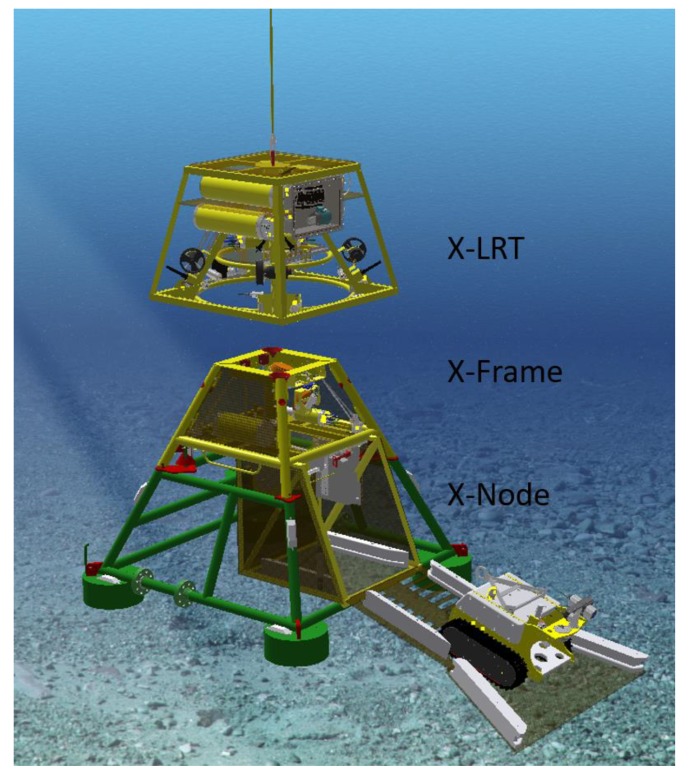
The bottom unit X-Net^®^ (Metas AS, Bergen, Norway) includes a bottom-based permanent cable connected docking station X-Node (green; see also Figure 2) and an instrumented exchangeable top unit (X-Frame; yellow) that can be launched and recovered with the Launch Recovery Tool (X-LRT). Note that the ARIM X-Frame includes the garage for the crawler (see Figure 2). This allows the user to operate and maintain the ARIM system without the assistance of expensive ROV vessel time.

The cabled observatory node system X-Net^®^ (Metas AS, Bergen, Norway) is composed of three main units: X-Node, X-Frame, and X-LRT (Launch and Recovery Tool; Figure 3). The X-Node is the permanent installed docking station connected directly to the infrastructure cable on the seabed. The X-Node is designed to receive an instrument platform with a garage module for the crawler. The instrument platform (X-Frame) is in principle the same as that used at LoVe but has been reconstructed and expanded to accommodate the garage module developed for Rossia. The front of the garage supports a ramp for the crawler to enter the garage from the seabed. The ramp also includes guiding for the crawler during on and off driving. During launch and recovery operations, the ramp is folded up, closing and protecting the crawler inside the garage. The garage holds a pin-less connector providing charging power for the crawler supported by the infrastructure when the crawler is docked in the garage. In case of power limitation from the infrastructure, the connector can provide charging power from a battery that is charged whenever power is available.

The garage has several operational cameras (see Table 1) and light installed for monitoring docking and cable winch during operation. Under autonomous operation (without umbilical), the pin-less connector can provide data communication between the infrastructure and the crawler when connected. Data, such as mission plans, can be transferred to the crawler before the mission start, and data collected by the crawler can be uploaded to the infrastructure after the crawler has docked after mission completion. During training of the autonomous piloting software, the operator can, via the infrastructure, communicate with the crawler through an optical fiber cable from a dedicated winch connecting the crawler to the garage. ARIM aims at autonomous operations including easy launch and recovery of the full system. An X-Frame/X-Node combination with automatic subsea interface where remotely operated wet mate-able connectors provide power and communication to the system has been developed. The connecting/disconnecting process of the X-Node can be done with a ROV or easier and cheaper with the tailored Launch and Recovery Tool (X-LRT; see Figure 3). Thus, the instrument platform with crawler can be launched and recovered without removing the permanent installed bottom X-Node infrastructure.

The X-LRT has the capacity to carry a payload of 6 tonnes supported by the wire to the ship. The unit is maneuverable through thrusters and the coupling–uncoupling and locking–unlocking processes are monitored with multiple cameras, ensuring correct positioning as well as correct mating of the connectors for power and communication. This project has bolstered the development of the new design of a garage module that fits into the existing X-Net^®^ (Metas AS, Bergen, Norway) infrastructure (see Figure 2).

### 3.3. Self-Sustained Operation with Fuel Cells

The flexibility and modularity of our system is supported by either power supply and communication at sites of cabled observatories (first step) or stand-alone operation using new fuel cell (FC) technology to power long-term operations (second step). To ensure efficient stand-alone operation, the energy system must carry enough power capacity to keep the sensors and the crawler in operation over an adequate time period. Further, the overall operation of the system must be safe and easy enough to ensure efficient launch and recovery.

**Figure 4:**
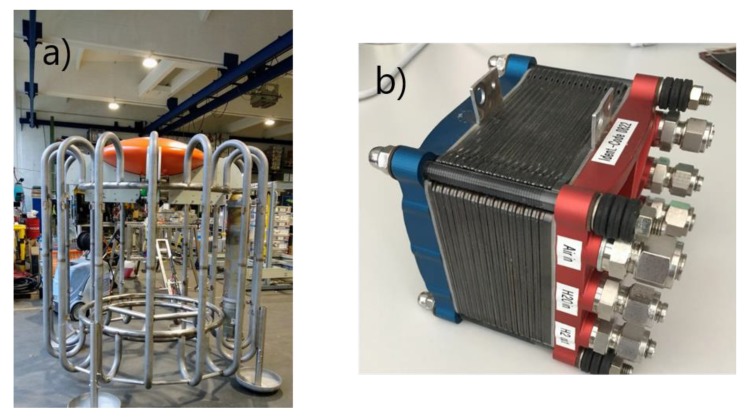
Long-term operation with a fuel cell and power storage system provides the basis for advanced marine research infrastructures. The frame on the left (**a**) shows the lander system that will carry the fuel cell components in the center with the gas bottles occupying the surrounding slots. On the right (**b**) is the membrane system of the fuel cell.

FC and rechargeable batteries are key technologies for 21st century marine research and monitoring systems. The long-term supply of energy in deep-sea research is currently facilitated via primary batteries with associated limitations and is not sustainable. This sparked the idea for the development of a deep-sea FC system for long-term monitoring approaches. Within this autonomous infrastructure, we built an FC system that provides about 160 kWh of energy and includes a 4 kWh storage system using lithium polymer (LiPo) battery technology (Figure 4). The fuel cell permanently delivers ~100–200 W.

Setup and operation of the FC are presently supported by ROV operations, but ROV-free operation will be possible in the future by using the new X-LRT illustrated in Figure 3. Using the LRT gives scientists and industrial operators more flexibility to move the complete system (X-Frame with crawler and the fuel cell power bank) to new locations without very expensive specialized ROV vessels.

### 3.4. Navigation and Piloting

Autonomous navigation and processing of images collected by the crawler is a core task in ARIM. To avoid fatal operational accidents due to failures in the autonomous navigation software, we used a tethered version during our first stage testing to navigate safely back to base, until the next fully autonomous tether-free generation vehicle is available. The autonomous control system is based on two major components:

1. The core element for autonomy is the simultaneous localization and mapping (SLAM) navigation algorithm, that creates the 2.5D bathymetry on the fly, using the SeaVision laser scanner plus the same map to localize itself (see Section 3.5);
2. A flexible mission execution layer based on Behavior Tree (BT) [33], that provides the functionality to act in response to events (e.g., payload sensor data) during the mission instead of following a fixed waypoint plan.

**Figure 5:**
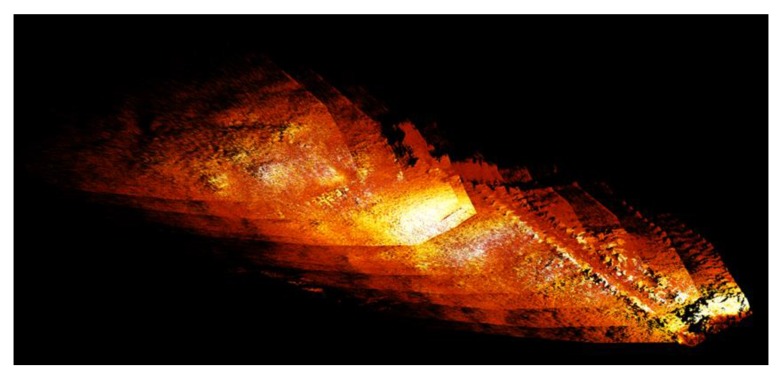
First results of a point cloud stably merging with the crawler odometry. Multiple scans were taken with a very slow stop-scan-move mission profile. The colorization shows the reflectivity of the seafloor to the laser line (remission). The data were acquired during a cruise on FS Alkor in November 2019, with the SeaVision system mounted on Rossia’s predecessor VIATOR from GEOMAR.

SLAM algorithms using point clouds from 3D laser scanners are quite common in terrestrial systems (see e.g., [34]), but these algorithms cannot be transferred to the marine environment directly. The main reason is the limited range of underwater scanning (attenuation, visibility, etc.), which results in comparably small point clouds. These in turn lead to limited overlapping areas between consecutive scans. This alone makes point cloud fusion problematic. The SLAM solution we propose uses several modalities to pre-align the point clouds, therefore allowing a faster fit with small overlap. The system uses the odometry of the crawler as the first motion estimation and then fuses it with a visual odometry from the SeaVision cameras using a Kalman filter. That estimation is then used as an input for a multi-hypothesis (e.g., Bayes or particle filter) point cloud fusion estimator. In a second step the point cloud is projected into a 2.5D bathymetry map. Figure 5 shows the first tests with real world data on the fusion between crawler odometry and multiple static point clouds.

The mission execution layer used in trials during November 2019, was based on “Behavior Tree.CPP”, a free and publicly available implementation of BT [35]. The BT kernel was attached to the action state automaton of the crawler and allowed to run missions. The next step will be integration of sensor information into the system to allow for reaction during a mission.

### 3.5. Automatic Data Acquisition and Processing for Ecosystem Functioning

**Figure 6:**
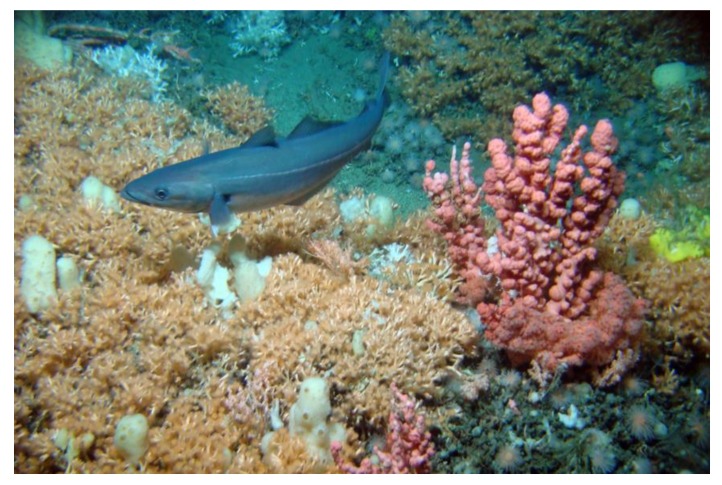
Coral reef with visible polyps. Their activity patterns in opening and closing inform about feeding dynamics. Further, activity patterns are also associated with human impacts (e.g., from industrial activities; Photo: S. Flögel).

The key sensor (see Table 1) of the benthic platform includes cameras as well as a 3D SeaVision scanner and bioacoustics package. Imaging assets at LoVe observatory (i.e., a camera mounted on node 1; see Figure 1) are used to track faunal changes at a coral reef habitat (Figure 6). By keeping the camera position fixed, changes in visual counts for individuals of different species over time can be efficiently obtained and thus uncover dynamics at all temporal scales (from hours to years). The images and the experiences from this project are now used in development of the ARIM image analysis.

**Figure 7:**
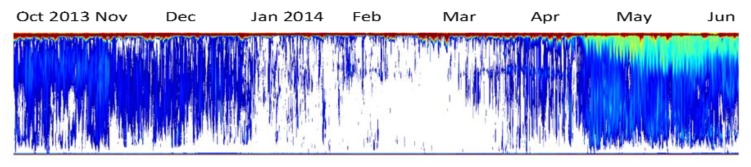
Seasonal dynamics of biomass in the water column (vertical axis) as observed in the acoustic records from October 2013 to June 2014 (horizontal time axis). The acoustic system also observes with seconds and cm resolution giving access to, for example, individual fish behavior information by depth (vertical axis). High biomass densities are defined by red color and biomass is gradually decreasing when the color goes towards blue. No biomass is recorded when the echogram is white (processed from LoVe data by E. Johnsen, Institute of Marine Research, Bergen).

At the same time, an acoustic package including EK-80 broadband scientific echosounder (Kongsberg ASA) with two multiplexed 70 kHz transducers (see Table 1) provide continuous monitoring of the water column (Figure 7). While the vertically pointing transducer is a reference for the vertical distribution and migration of organisms up to 1000 m above (dependent from frequency), the horizontal moveable transducer enables a pelagic habitat search, covering a sector from the bottom to the surface with range up to 1000 m. Backscattering from planktonic organisms to whales is recorded at any time interval from seconds to seasons [7,36]. Autonomous operation, including feedback from the acoustic backscatter to the operation software, enables detection of behavioral-related dynamics of organisms. With an adequate ping rate the acoustic monitoring can resolve individual behavior to a scale of mm and seconds [7]. This includes tracking the behavior of individual organisms as well as the crawler.

In parallel with the acoustic monitoring, the automated video tracking and classification have provided information on the dynamics of benthic megafauna in space and time. The detailed dynamics obtained through the coral reef images (see Figure 6) have helped in understanding temporal dynamics of polyps while the acoustic data have provided a better understanding of timing of ecosystem processes as well the impact of human activities ([37]; Figure 6). Such studies are important for the development of automatic image processing software for categorization and identification of species as well as of processes.

**Figure 8:**
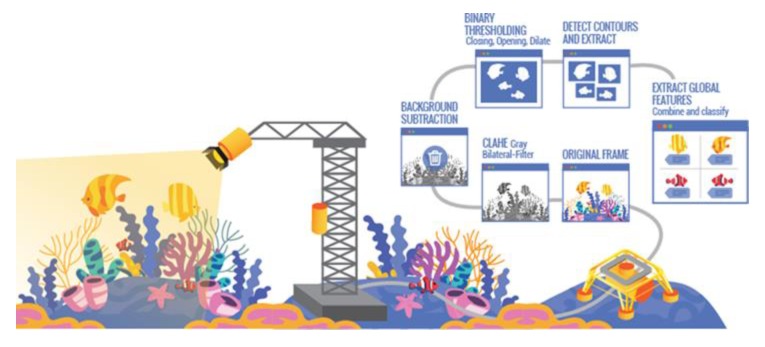
Designed automatic image processing pipeline for HD imaging that could also be applied to acoustic outputs.

One of the core ideas of our approach is to establish a real-time data processing and interpretation system that enables categorization and, when possible, species identification in near real time both from the imaging and the acoustic sensors. An image processing pipeline was developed based on computer vision and machine learning tools, for detecting, segmenting, and classifying underwater animals from the dataset collected by the LoVe observatory (Figure 8). First, these images are resized to speed up the process; then several techniques are applied to improve the contrast between the background and the species to be detected. Afterwards, the background is subtracted, followed by the application of filters to segment the contours correctly. Finally, once these contours are extracted, different global characteristics are extracted from each cropped image and combined, and ultimately classified. Temporal variation in images at LoVe is demonstrated in the video presentation of image data set from LoVe (Supplementary Video S1). With stereo camera images we were able to establish 3D images of objects and this supports the identification of component characteristics (Supplementary Video S2).

**Figure 9:**
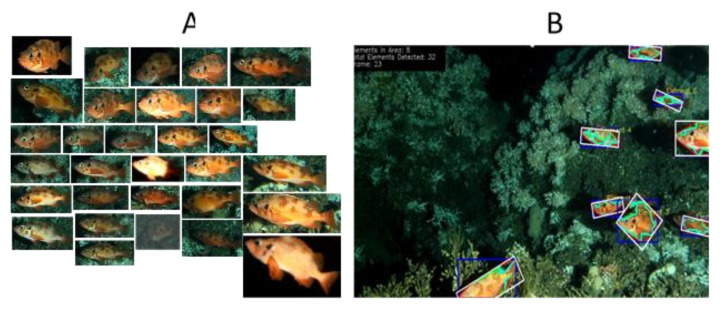
(**A**) A set of images from a larger training library as an example of supervised tracking and classification of Rockfish (*Sebastes* sp.) within a constant field of view from archived videos at LoVe. (**B**) Results of automated tracking and counting of individuals passing by, as evidenced in a video by the recognition boundary boxes (see Supplementary Video S1).

Fortunately, processing software for image interpretation improves continuously [38,39], and we are now calibrating our analysis from images’ databases containing identified objects. A computationally demanding function of an underwater video monitoring system is the capability to automatically track and classify animals within different species as pre-established categories, based on pattern recognition methods. Once pattern recognition has taken place, it is necessary to develop different methods of supervised classification based on training sets (Figure 9) to associate them with their corresponding species and validate the performances of the different routines according to the image quality (Figure 7; see also Supplementary Video S2).

This pipeline is now being adapted for crawler images. This adjustment is needed as the environment will become more challenging. The advancing crawler creates a moving background and reduces visibility due to the turbidity caused by the sand cloud in the wake of the crawler, as well as by normal sediments/rocky background.

Above we described the development of various components and tailoring of a complete novel monitoring system that is now under testing and completion. The status, challenges, and expected time for realistic full-scale testing is summarized in Table 2. The crawler will then perform transect analyses along the seafloor using its full set of sensor technologies including the SeaVision 3D camera, which quantifies volume changes of habitats forming megafauna. Similar to the bioacoustics sensor operations on the X-Net, the crawler operations will then drastically expand the study/monitoring site from m^2^ to km^2^.

## 4. Discussion

In this paper, we merged technologies and competence with the overarching goal to meet future requirements in marine research and monitoring. We experienced that multidisciplinary engineering and ecological expertise can efficiently respond to challenges that would be unsolvable individually [22]. With our technological advances we demonstrate that the hardware and software components from various partners can be merged, and preliminary results demonstrate the technology’s potential. The next step is to carry out pilot monitoring activities where all technological components (i.e., node, crawler, and fuel cell) operate together under full autonomy.

Our technology collects key information of ecosystem components and their interaction and dynamics. The urgent need for such key information from the marine environment, and associated tools for filling critical knowledge and information gaps, is obvious from the United Nations (UN) 2017 proclamation of “An International Decade of Ocean Science for Sustainable Development” [41], based on recommendation from the Intergovernmental Oceanographic Commission (IOC) [42]. The basic need for adequate time–space information of key ecosystem components cannot be satisfied through conventional methodologies due to cost and capacity limitations associated with using available research vessel capacity [7]. Further, few available approaches allow a combination of pelagic and demersal sampling simultaneously. We think that the ARIM development scenario is a promising approach to making adequate sampling available to scientists and might thus be an important tool for fulfilment of the goals launched by the UN.

ARIM’s performance is totally dependent on a cross-disciplinary team of scientists and engineers working together towards a common goal. The team aims at continuing the development and utilizing the experiences of partners in ongoing projects at existing observatories (Neptune and LoVe). A combination of acoustics with oceanography as done by Engeland and colleagues [43] demonstrates the strength of coordinated multiparametric sampling in time and space with various sensors, with imaging at the center of that development for ecological monitoring (e.g., [44]). The unique contribution of ARIM is to establish technology and routines for combining imaging techniques of the benthic habitat and acoustic sampling of the pelagic habitat. This is a key factor for understanding marine ecosystem variability in time and space. Massive recurring population movement rhythms into the water column and across seabed depth ranges facilitate the exchange of energy among different marine compartments at rates faster than mere oceanographic conditionings, thus making animals efficient carbon carriers both as predators and preys, as well as when defecating [6]. Synchronization of biological activity in deep-sea benthos may also occur in relation to day-night cycles in an indirect fashion; i.e., mediated by the intermittent presence and absence of deep-scattering layers of predators and preys, rhythmically appearing within the benthic boundary layer over a 24-h period (e.g., [44]). Such rhythmic dynamics may also be accompanied by not yet quantified changes in background illumination at the seabed caused by bioluminescence of the scattering layers [45]. If such rhythms influence species relative abundances and community composition towards the continental margins where observatory networks are deployed, a temporal variability in measured ecological indicators (species abundance, biomass as well as biodiversity) must be expected [4].

We expect that an entirely new class of moving robotic platforms, spanning from tethered and untethered crawlers with a suite of advanced sensors, will support a dramatic improvement in deep-sea monitoring [5]. Such a technological development will favor a cross-over with other monitoring networks of ecological relevance, merging Eulerian and Lagrangian approaches [4]. Cabled observatories and their docked crawlers will increasingly integrate the capability for communication with other technological assets, such as neutrino telescopes with their vertically-extended moored profilers, ARGO floats, and even animal-borne sensors.

Envisaged autonomous robotic exploration and monitoring technologies are being developed using space analogs on Earth [46,47]. Marine observatory deployment scenarios are considered operative analogues of extraterrestrial oceans exploration, testing for increased reliability in robot autonomy in self-assembling, -repair, and -energetic tasks [17]. These aspects are of broad relevance for planetary surface explorations [48], where robotic platforms will subsidize humans in space exploration over the next decades [49]. Lagrangian exploration approaches, such as the one ARIM action envisages, should be used as a test bed for the tailoring of solutions adapted to explore exo-oceans on, for example, the Enceladus moon of Saturn [34].

Automatic processing and interpretation of both images and acoustics are still under development and will require substantial input in the years to come to produce reliable results [20]. Although the technique is promising, we still see major challenges in establishing fully automatic systems that can operate unattended over extended periods. Energy will restrict sampling density, and hence, temporal gaps may obscure processes that otherwise would have been detected. The energy limitations are continuously improved through better batteries and more power efficient fuel cells and sensing systems. Reliable imaging techniques require long-term operation with cable collections through an online observatory to ensure that data are properly validated and that recognition algorithms are updated accordingly [10].

## Figures and Tables

**Table 1:** The main environmental sensors assets used for ecological monitoring as pursued by ARIM.

Sensors	Type/Model	Crawler	X-Frame
Current profiler long range (m/s and °)	Acoustic current meter/Nortek Signature 250		X
Current profiler short range (m/s and °)	Acoustic current meter/Nortek Aquadop	X	X
Pressure (dbar)	Xylem AADI 4117D *		*
Temperature (°C)	Xylem AADI 4117D *		*
Salinity (°/_oo_)	Xylem AADI 4319A *		*
CTD (°C, dBar, °/_oo_)	ADM CTD	X	
Oxygen (µmol/L))	AADI optode 4531	X	
Turbidity (FTU)	Seapoint turbidity meter	X	*
Chlorophyll (μg/L)	Seapoint fluorometer	X	
High-definition (HD) imaging		X	
Operational cameras	3 Metas web-cameras (1 UWC-960, 2 UWC-210)		X
Acoustic imaging	Simrad WBT-mini with ES-70 CD and ES-200 CD transducers		X
Ambient noise	Ocean Sonics SB-35 ETH		X
Laser Scanning	Kraken SeaVision^®^	X	

* Optional sensors that can be installed in the interface container lid.

**Table 2:** Status, expected challenges during completion, and expected time of first full-scale testing.

Element	Status	Challenges	Expected Completion	Comment
Crawler system	Tested at shallow water	Garage–crawler symbiosis and cable operation	First test mid-2020	Similar crawlers have been produced and given valuable experience for the completion of the ARIM crawler.
The bottom-based system	Platform tested at LoVe and at a commercial oil production site	Integrating garage with charging and cable winch for the crawler. Combine these functions and X-Frame as a sensor carrier	First field tests with complete system mid-2020	The optical fibre cable on the winch is used for training the navigation software prior to full autonomy operations
Self-sustained fuel cell	Design and laboratory version completed. Construction of frame and recovery system finished	Integration of fuel cell within the ARIM system. Establish operational routines	First field tests with complete system mid-2020	This module is presently an add-on to the ARIM platform. We expect a full integration in the future
Navigation and piloting	Mainly laboratory test. First field test in shallow water in 2019	Deep water operation in unknown location	Tests under realistic operational conditions mid-2020	This software will be under continuous development based on experience and on development in a fast-developing software field
Automatic data acquisition and processing	Large amount of data from LoVe used to train the system. All routines are operated according to specifications	Evaluation of system capacity in handle changing visibility and routines capability to work at different habitat conditions	Tests under realistic operational conditions mid-2020	Changing habitat conditions like light and turbidity affect visibility. This is a general challenge in marine imaging that requires attention. The complete processing pipeline as well as the associated problems and future challenges is detailed in [40]

## Supplementary Materials

The following are available online at https://www.mdpi.com/1424-8220/20/6/1614/s1, Video S1. Demonstrates a typical data set from the LoVe coral reef that is used to train the AI identification algorithms. Video S2. Establishing 3D images from stereo images.
